# Relationships Among Personality, Daily Speaking Voice Use, and Phonotrauma in Adult Female Singers

**DOI:** 10.1044/2021_JSLHR-21-00274

**Published:** 2021-11-03

**Authors:** Laura E. Toles, Nelson Roy, Stephanie Sogg, Katherine L. Marks, Andrew J. Ortiz, Annie B. Fox, Daryush D. Mehta, Robert E. Hillman

**Affiliations:** a Massachusetts General Hospital, Boston; b MGH Institute of Health Professions, Boston, MA; c The University of Utah, Salt Lake City; d Harvard Medical School, Boston, MA

## Abstract

**Purpose:**

This study sought to determine whether personality traits related to extraversion and impulsivity are more strongly associated with singers with nodules compared to vocally healthy singers and to understand the relationship between personality and the types of daily speaking voice use.

**Method:**

Weeklong ambulatory voice recordings and personality inventories were obtained for 47 female singers with nodules and 47 vocally healthy female singers. Paired *t* tests investigated trait differences between groups. Relationships between traits and weeklong speaking voice measures (vocal dose, sound pressure level [SPL], neck surface acceleration magnitude [NSAM], fundamental frequency, cepstral peak prominence [CPP], and the ratio of the first two harmonic magnitudes [*H*_1_ *–H*_2_]) were examined using pairwise Pearson *r* coefficients. Multiple regressions were performed to estimate voice parameters that correlated with two or more traits.

**Results:**

Singers with nodules scored higher on the Social Potency scale (reflecting a tendency toward social dominance) and lower on the Control scale (reflecting impulsivity) compared to the vocally healthy singers. In vocally healthy singers, vocal dose measures were positively correlated with a combination of Wellbeing (i.e., happiness) and Social Potency, mean SPL was positively correlated with Wellbeing, SPL variability was positively correlated with Social Potency and negatively with Harm Avoidance, and CPP mean was positively correlated with Wellbeing. Singers with nodules had a negative correlation between NSAM skewness and Social Potency. Both groups had negative correlations between *H*_1_ *–H*_2_ mean and Social Potency and Social Closeness.

**Conclusions:**

Singers with nodules are more socially dominant and impulsive than vocally healthy singers. Personality traits are related to daily speaking voice use, particularly in vocally healthy singers. Individuals with higher levels of traits related to happiness and social dominance and lower Harm Avoidance tended to speak more, with higher laryngeal forces, with more SPL variability, and with more pressed glottal closure, which could increase risk of phonotrauma.

Singers comprise between 11% and 29% of the patient population in voice clinics (Kridgen, 2019; Titze et al., 1997), presumably due to the increased risk that accompanies the heavy vocal requirements of singing and the implication vocal health has on their livelihood (Pestana et al., 2017; Phyland, 2017; Phyland et al., 1999). Most common among singers are disorders associated with vocal hyperfunction (Verdolini & Ramig, 2001), which can be broadly defined as excessive or imbalanced muscular forces employed to produce voice (Hillman et al., 1989, 2020; Oates & Winkworth, 2008). Like individuals in other high voice use occupations, singers have an increased risk of vocal hyperfunction causing damage to vocal fold tissue (e.g., vocal nodules; Guss et al., 2014; Roy et al., 2004), referred to as phonotraumatic vocal hyperfunction (PVH; Hillman et al., 2020). There is a relative consensus that phonotrauma can stem from cumulative vocal fold tissue damage and/or reaction to continuous tissue inflammation (Mehta et al., 2012; Titze et al., 2003). The higher risk of phonotrauma in singers is commonly assumed to be due to the increased vocal demands associated with singing (e.g., sustained high intensity throughout a wide pitch range). Unfortunately, singing demands alone do not explain why some singers develop phonotraumatic lesions whereas other singers within the same genre, with similar training, and with comparable occupational/singing vocal demands do not.

The nature of the singer's career may lend itself to situations that also place heavy demands on the speaking voice, such as social activities with fellow singers, speaking before or after performances in noisy environments such as restaurants or bars, or part-time jobs that require speaking extensively and/or over background noise. A recent study that monitored the voice use of healthy female college student singers for 1 week found that singers used their voices for speaking 3 times more than for singing (Toles et al., 2020). A follow-up study determined that singers with phonotrauma spent significantly more time speaking, but not more time singing, during a typical week than singers with healthy voices (Toles et al., 2021). In fact, the investigators found that singers with healthy voices tended to spend more time singing during a typical week as compared with singers diagnosed with phonotrauma. This study also found that singers with phonotrauma have a tendency to speak with higher-than-average laryngeal forces (e.g., negative skewness of the sound pressure level [SPL] and neck surface acceleration magnitude [NSAM] distributions) and more abrupt/pressed glottal closure (e.g., more restricted variability toward higher values in the ratio of the first and second harmonic magnitudes [*H*_1_ *–H*_2_]) in daily life—which is in agreement with other recent studies that monitored the total phonation (singing and speaking combined) of both singers and nonsingers with PVH for an entire week (Van Stan, Mehta, Ortiz, Burns, Marks, Toles, Stadelman-Cohen, et al., 2020; Van Stan, Mehta, Ortiz, Burns, Marks, Toles, Vangel, et al., 2020). Toles et al. (2021) hypothesized that increased speaking voice use, combined with the tendency to speak with higher laryngeal forces and more abrupt/pressed glottal closure (suggesting the more frequent use of louder voice), might represent etiological factors that are influenced by the intrinsic personality dispositions of singers who develop phonotrauma.

Personality can be described as the distinctive ways in which an individual behaves, feels, or thinks over time and across situations (Twenge & Campbell, 2016). Personality traits have been found to be associated with the presence of hyperfunctional voice disorders (Roy & Bless, 2000; Roy et al., 2000a, 2000b). Most theoretical models of personality are based on a foundational assumption that personality is composed of various characteristics or traits, which are largely stable over time, though continued development of personality over the life span is possible (Haan et al., 1986; Roberts & Mroczek, 2008; Scollon & Diener, 2006). A trait-based approach, which has been prominent in modern literature because it facilitates comparisons across people, consists of higher order personality traits (e.g., Extraversion and Neuroticism; Botwin & Buss, 1989; Conley, 1985; Field & Millsap, 1991; John, 1990), each of which consists of a number of lower order characteristics or facets (Goldberg, 1990). Higher order traits have been found to be robust predictors of various types of human behavior, and facets tend to be even stronger predictors of behavior (Costa & McCrae, 1992, 1995).

The Trait Theory of Voice Disorders (TTVD; Roy & Bless, 2000; Roy et al., 2000b), which has proposed that particular types of personalities are associated with PVH, integrates a trait-based approach with biological models of personality, developed by Eysenck and Gray, that examine whether traits have a biological basis (Eysenck & Levey, 1972; Gray, 1981). These biological models suggest that individuals who are more extraverted tend to be more sociable, dominant, active, and less sensitive to stimulation than introverts (Eysenck & Levey, 1972; Matthews & Gilliland, 1999; Stenberg et al., 1990). Such tendencies theoretically lead the extravert to seek out external stimulation through active/approach behaviors (Schmeck & Lockhart, 1983), whereas introverts tend to inhibit behavior due to reactions to punishment, nonreward, and threat (Gray, 1981). Neuroticism is thought to reflect the reactivity of the arousal system (i.e., a lower threshold for experiencing negative emotions results in increases in emotional arousal) and interacts with extraversion-related tendencies (Fowles, 1980). For instance, an individual who is extraverted will demonstrate an increase in active/approach behaviors if they also have high levels of Neuroticism (Newman & Wallace, 1993; Wallace et al., 1991). These traits have implications on motor behavior, which could theoretically lead to distinct patterns of vocal hyperfunction (Hillman et al., 2020).

The TTVD framework posits differing characterological etiologies for PVH and nonphonotraumatic vocal hyperfunction (NPVH; Roy & Bless, 2000; Roy et al., 2000a, 2000b). According to this model, those with PVH, who have been found to be highly extraverted and moderately neurotic, are more likely to be sensitive to social reward, resulting in a tendency toward vocally traumatic behaviors (e.g., they are more likely to seek out situations in which they are surrounded by friends in a stimulating environment, which leads to talking loudly or excessively; Roy & Bless, 2000; Roy et al., 2000a, 2000b). To investigate the relationships between specific facets and voice disorders, Roy and colleagues used the Multidimensional Personality Questionnaire, which has a higher order structure roughly similar (though not identical) to Eysenck's model, but also measures facets (Tellegen & Waller, 2008). Females with vocal nodules tended to have high levels of Social Potency (those who enjoy being noticed, dominant) and low levels of Control (those who are impulsive; Roy et al., 2000a). An expanding number of studies have used the TTVD framework as a foundation for subsequent research. Several studies have found that personality type and its relation to stress is associated with the presence of hyperfunctional laryngeal muscle patterns, but these studies have been conducted in laboratory settings mostly in healthy individuals (Dietrich et al., 2008, 2012; Dietrich & Verdolini Abbott, 2012; Helou et al., 2013, 2018, 2020). There is a scarcity of information regarding the influence of personality on voice use in “real-world” situations that occur outside of a clinical or lab setting.

Furthermore, the personality dispositions/traits of singers have been sparsely addressed in the literature. Among existing research, healthy singers have only been analyzed as a small group compared to other types of musicians (e.g., string players; Butkovic et al., 2015; Kemp, 1981, 1996; Miksza, 2006). Studies have found that singers have higher levels of Extraversion and Self-Confidence (Heller et al., 2015; Kemp, 1981, 1996), higher levels of Independence and Sensitivity (Kemp, 1981), and higher levels of Neuroticism (Heller et al., 2015) than other types of musicians. No studies have specifically investigated the personality traits of singers with dysphonia. In fact, studies establishing the framework of the TTVD (Roy & Bless, 2000; Roy et al., 2000a, 2000b) did not address singers, likely because of the supposition that singers are more likely to develop phonotrauma from repetitive trauma to the vocal folds due to singing voice use rather than also involving psychosocial factors. These gaps in the literature have implications for our collective understanding of an extremely high-risk and high treatment-seeking population. Filling these gaps could influence clinical and therapeutic decision making and potentially refine our ability to identify those who are at higher risk for developing phonotrauma.

Development of phonotrauma in singers likely stems from a multidimensional etiology since no single factor has been found to explain the phenomenon that some singers develop phonotraumatic lesions whereas others with similar voice demands do not. The aims of this study were (a) to determine whether personality traits related to extraversion, neuroticism, and impulsivity are more strongly associated with singers who have nodules as compared to a matched group of singers with healthy voices and (b) to understand the relationship between personality traits and the types of daily speaking voice use that could lead to phonotrauma in singers. We hypothesized that singers with phonotrauma would demonstrate higher levels of traits related to extraversion, neuroticism, and impulsivity than singers with healthy voices and that these traits will be associated with the types of daily speaking voice use that are considered vocally traumatic (e.g., higher vocal doses and speaking louder/with higher laryngeal forces).

## Method

### Participants

This study protocol was approved by the institutional review board at the Massachusetts General Hospital. Forty-seven female individuals with a diagnosis of bilateral vocal fold nodules who self-identified as singers and 47 matched control singers with a healthy vocal status were analyzed in this study. Only female individuals were included in the analysis because of their substantially higher incidence of vocal fold nodules (Zhukhovitskaya et al., 2015). Singers with vocal fold nodules were recruited through a convenience sample from the Massachusetts General Hospital Center for Laryngeal Surgery and Voice Rehabilitation after the diagnosis was confirmed by a laryngologist using videostroboscopy and corroborated by a voice-specialized speech-language pathologist (SLP). In addition to the diagnosis of bilateral vocal fold nodules, all patients enrolled in the study had to meet the following criteria: (a) have self-reported current voice difficulty in the speaking and/or singing voice; (b) were between the ages of 18 and 65 years; and (c) had self-identified as a singer in a professional, semiprofessional, student, or teacher capacity. Patients were excluded if they were currently participating in or had recently concluded a course of voice therapy or if they had a history of laryngeal surgery.

Each singer with vocal fold nodules was matched with a control participant who was a singer with a healthy voice. To ensure the two groups were as similar to one another as possible, each vocally healthy singer was closely matched to each singer with vocal fold nodules based on age (± 5 years), singing career level (professional, semiprofessional, student, or music/voice teacher), and primary singing genre (classical or nonclassical). Control participants were recruited through snowball sampling, as enrolled patients identified a person who would be an appropriate match for them based on the above criteria. For those unable to find a suitable match, convenience sampling was used (e.g., e-mails to local music conservatories). Potential control participants were screened by an SLP using the following criteria: (a) no current or previous voice problems, (b) have normal perceptual voice quality, (c) are between the ages of 18 and 65 years, (d) have healthy laryngeal status confirmed through videostroboscopy, and (e) have typical hearing confirmed through a pure-tone hearing screening (25 dB HL at 0.5, 1, and 2 kHz).

An auditory-perceptual voice analysis was conducted by a voice-specialized SLP for each singer with vocal fold nodules using the overall severity rating of the Consensus Auditory-Perceptual Evaluation of Voice (CAPE-V; Kempster et al., 2009), which is a 100-point visual analog scale with higher scores reflecting higher levels of dysphonia. The mean (standard deviation [*SD*]) of the overall severity rating for this group was 21.45 (13.31). The CAPE-V scores were not included in the statistical analysis and are only provided for descriptive purposes. All participants provided self-report ratings for the Voice-Related Quality of Life (V-RQOL; Hogikyan & Sethuraman, 1999) and the Singing Voice Handicap Index (SVHI-10; S. M. Cohen et al., 2009) through REDCap electronic data capture tools hosted at the Mass General Brigham health system (Harris et al., 2009, 2019). Briefly, lower voice-related handicap is reflected through higher V-RQOL scores and lower SVHI-10 scores. These results are presented in Table 1.

**Table 1.** Group-based demographic information including mean (standard deviation) of age, sum of participants in each primary singing genre and singing career level, and mean (standard deviation) for Voice-Related Quality of Life and Singing Voice Handicap Index-10.

Parameter	Vocally healthy singers	Singers with nodules
Age (years)	22.5 (4.8)	21.4 (4.3)
Singing genre		
Classical	8	8
Nonclassical	39	39
Singing career level		
Professional	3	3
Semiprofessional	5	5
Music/voice teacher	3	3
Student	36	36
Voice-Related Quality of Life		
Social–emotional score	95.5 (6.7)	66.4 (24.8)
Physical functioning score	95.2 (5.8)	67.8 (21.5)
Total score	95.3 (5.4)	67.2 (20.7)
Singing Voice Handicap Index-10		
Total score	6.2 (4.6)	20.2 (8.8)

### Data Collection

Each participant completed a personality survey (described below) and were asked to undergo 7 days of ambulatory voice monitoring. For the participants diagnosed with vocal fold nodules, this assessment phase took place prior to initiation of any intervention for their vocal fold pathology (i.e., voice therapy or laryngeal surgery). Participants were assessed during a week that included typical voice use (e.g., student singers were monitored when school was in session). Of note, 11 of the 47 vocally healthy singers were enrolled and assessed during the phased reopening process associated with the COVID-19 pandemic. All 47 singers with nodules were enrolled and assessed prior to the beginning of the pandemic.

### Personality Inventory

The Multidimensional Personality Questionnaire–Brief Form (MPQbf) was administered to each participant through REDCap. This scale was deemed to be the most appropriate self-report questionnaire to fit our conceptual framework because it measures higher order traits that are similar to Eysenck's trait constructs and also measures trait facets in ways that are interpretable. The higher order traits that the MPQbf measures include Positive Emotionality (PEM; conceptually related to Extraversion), Negative Emotionality (NEM; conceptually related to Neuroticism), and Constraint (CON; Patrick et al., 2002; Tellegen & Waller, 2008). CON is inversely related to the concept of impulsivity. Both PEM and NEM are hypothesized to be temperamental in nature and account for dispositions toward positive and negative emotions, respectively (Patrick et al., 2002). Those with high PEM scores are more likely to experience a lower threshold for positive emotional experiences. High NEM scores would suggest a lower threshold for experiencing negatively valenced emotions (Tellegen & Waller, 2008).

The MPQbf includes 11 subscales that measure individual facets that comprise the higher order traits (Patrick et al., 2002). The facets that comprise the PEM scale (and what high scores represent) are Wellbeing (happy disposition, pursues fun activities, and optimistic), Social Potency (dominant, persuasive, and leader), Achievement (ambitious, enjoys challenge, hard-working, and perfectionist), and Social Closeness (affectionate, sociable, and values close relationships). The facets comprising NEM include Stress Reaction (nervous/tense, sensitive, anxious, and easily upset), Alienation (tendency to feel betrayed, exploited, and unlucky), and Aggression (violent, vengeful, and victimizes others for own gain). The CON scale is composed of the following facets: Control (cautious, reflective, anticipates events, and inverse of impulsive), Harm Avoidance (avoids risk of injury, dislikes risky adventures, and motivated by fear), and Traditionalism (endorses religion, condemns selfishness, and advocates high moral standards). One lower order trait scale that does not load onto any of the three higher order scales is Absorption (vivid imagination, engrossed in own thoughts, and thinks in images; Patrick et al., 2002). Appendix A provides detailed descriptions of each of the traits and facets. The MPQbf was used with permission from the Minnesota Press.

### Ambulatory Data Collection

Each participant completed 7 days of ambulatory voice monitoring using a small accelerometer that attaches to the anterior neck (just below the thyroid prominence) connected via a thin wire to a smartphone-based recording system. Participants underwent an in-lab educational visit at the beginning of their monitoring period, during which they were instructed how to place the sensor consistently each day. As described in several previous publications, the Voice Health Monitor smartphone application guided participants through a procedure that enabled SPL calibration (dB SPL) of the accelerometer (Švec et al., 2005) each morning (Mehta et al., 2012, 2015; Van Stan et al., 2015; Van Stan, Mehta, Ortiz, Burns, Marks, Toles, Vangel, et al., 2020). This was accomplished by simultaneously recording the oral acoustic output with a digital voice audio recorder (H1 Handy Recorder, Zoom Corporation) positioned 15 cm from the lips as participants sustained an /ɑ/ sound gliding on a steady pitch from loud to soft voice production.

The ambulatory data that were collected included was a raw acceleration waveform DAT file of the participant's neck surface vibration, sampled at 11,025 samples per second and 16 bits, for each of the days monitored (Mehta et al., 2012), which was processed using customized MATLAB scripts. As described in previous publications (Mehta et al., 2012, 2015), the method used to distinguish between voiced and nonvoiced activity involved extracting measures of fundamental frequency (*f_o_*) and SPL from nonoverlapping 50-ms frames. Frames were considered voiced activity if the following thresholds were met: (a) SPL greater than 45 dB SPL, (b) *f_o_* between 70 and 1000 Hz, (c) the first nonzero-lag peak in the normalized autocorrelation greater than .6, and (d) the ratio of low- to high-frequency energy exceeding 22 dB.

Two discrete Fourier transforms were computed in succession with a logarithmic transformation between them to extract cepstral peak prominence (CPP) from the raw acceleration signal. CPP was defined as the difference between the magnitude of the highest peak and the baseline regression level in the power cepstrum. The peak search was limited to quefrencies between 2.4 and 12.0 ms, which corresponds to frequencies of 416.7 and 83.3 Hz, respectively (Mehta et al., 2015, 2016). *H*_1_ *–H*_2_, which is a measure that represents the difference between magnitudes (in dB) of the first and second harmonics in the frequency spectrum, was derived from a 1,024-point fast Fourier transform of each 50-ms voiced frame (Mehta et al., 2019).

Three average vocal dose measures were computed, which included (a) time dose (accumulated phonation time), calculated as the percentage of phonation during the total time the participant was monitored; (b) cycle dose, which estimated the number of vocal fold oscillations during the total monitoring time and normalized based on hours monitored (cycles per hour); and (c) distance dose, which estimated the cumulative distance traveled by the vocal folds during the total monitoring time and is calculated using estimates of cycle dose with estimates of vocal fold vibratory amplitude based on SPL and normalized by hours monitored (Mehta et al., 2015; Titze et al., 2003).

Frames of the raw acceleration signal were classified as containing singing or speech using an objective singing classifier (Ortiz et al., 2019; Toles et al., 2020, 2021). To address our research aims, only voiced frames classified as speech were analyzed. Weekly distributional statistics for SPL, *f_o_*, CPP, and *H*_1_ *–H*_2_ included mean (mode for *f_o_*), *SD*, minimum, maximum, range, skewness, and kurtosis values. To avoid including nonvoiced outliers in the values associated with the range of the weeklong ambulatory voice recordings, we report the 5th percentile values as “minimum,” the 95th percentile values as “maximum,” and the 5th to 95th percentiles as “range.”

### Statistical Analysis

The raw score of each of the MPQbf trait and facet scales was transformed into a standardized *T* score based on the population mean and *SD* (Patrick et al., 2002). Scores of 50 represent the normalized mean with ± 10 points representing 1 *SD*. For each higher order trait and each trait facet, a paired-samples *t* test was conducted to determine group differences. A paired-samples test was chosen over an independent-samples test to maintain the close patient–control pairing. A family-wise error rate correction was applied to the alpha level of significance; therefore, an adjustment was made based on how many tests were conducted within each trait family (e.g., the PEM family, which includes one higher order trait and four facets, was corrected by dividing the α level of .05 by five, yielding .01). The effect size for each difference was calculated using Cohen's *d* values for paired differences (mean difference divided by the *SD* of the difference) and interpreted as (small ≤ 0.19, small-to-medium = 0.20–0.49, medium-to-large = 0.50–0.79, and large ≥ 0.80; J. Cohen, 1988).

Next, we conducted two separate stepwise binary logistic regression models to determine which traits, or combinations of traits, could accurately discriminate between singers with nodules and singers with healthy voices. One model was conducted using the 11 trait facets as the predictors. To avoid multicollinearity between trait facets and higher order traits, a separate logistic regression model was conducted using the three higher order traits. For each logistic regression model, we calculated the overall classification accuracy of the model, the area under the receiver operating characteristics curve (AUC), and the odds ratio (OR) for each predictor variable.

To address our second research question, we examined the pairwise relationships between personality and voice measures in each group separately using Pearson *r* correlation coefficients. The strength of the correlation was determined using the conventional interpretation of Pearson *r* for social sciences (small = .1, medium = .3, large = .5; J. Cohen, 1988). Then, to identify which of the traits are the most highly associated with vocal behavior in each group, we completed a multiple regression model for each voice outcome that demonstrated a statistically significant correlation (using a cutoff of *p* ≤ .05) with at least two higher order traits or two trait facets based on the group-specific pairwise correlation results. The dependent variable for each model was the voice outcome measure, and the independent variables included the higher order traits or facets that were significantly correlated with the voice outcome in the pairwise correlation stage of analysis. Higher order traits were not entered into the same regression models as the trait facets because the facets comprise the higher order traits. For each multiple regression model, we interpreted the overall significance of the model using the multiple *R* and the model *R* ^2^. Then, we identified the relative contributions of each trait/facet using the standardized β coefficients, significance level, and partial *r* ^2^ (i.e., the amount of unique variance attributed to each trait/facet).

A series of regression diagnostics was conducted to assess whether the data met the assumptions for parametric analysis. First, the distributions of both the personality and the outcome variables were visualized. There was moderate-to-large negative skew in the distributions for PEM and its associated facets in the patient group and a negatively skewed distribution for Social Potency in the control group. To determine whether the skewed distribution of these predictor variables violated the assumption of linearity, we conducted regression diagnostics using a separate simple regression model for each personality variable with a skew value of larger than |1|. All the models met the assumption of linearity and homoscedasticity, with normally distributed residuals. Durbin-Watson tests were conducted to determine if there were any correlations among the residuals, and all models had acceptable values (Durbin-Watson values of > 1 and < 3). Therefore, we moved forward with our planned parametric analysis.

## Results

Demographic information (i.e., age, singing style, and professional singing level) and self-assessed V-RQOL and SVHI-10 scores for the study sample are presented in Table 1. Results for the paired-samples *t* tests that assessed differences between singers with nodules and singers with healthy voices in higher-order personality traits and trait facets are presented in Table 2, along with the mean and *SD* of the standardized *T* score for each group. The two facets that showed a statistically significant difference between groups at the corrected significance level were Social Potency (singers with nodules showing higher levels; *p =* .001, *d* = 0.49) and Control (singers with nodules showing lower levels; *p* = .004, *d* = 0.43). The higher-order trait of PEM and the trait facet of Social Closeness both approached the corrected significance level (*p* = .016 and .041, respectively), which showed the singers with nodules having higher levels in both traits.

**Table 2.** Means (standard deviations) of standardized *T* scores of personality higher-order traits and lower-order facets and paired *t*-test results (group difference means and standard deviations, 95% confidence interval [CI] for the mean difference, *t* statistic, significance value, and Cohen's *d* effect sizes) for differences between singers with nodules and matched control singers with healthy voices (47 matched pairs).

Personality trait	Vocally healthy singers	Singers with nodules	Group difference	95% CI	*t*	*p*	*d*
Positive Emotionality	55.7 (8.1)	60.5 (9.5)	−4.87 (13.08)	[−8.93, −.82]	−2.5	.016	0.36
Wellbeing	50.9 (9.9)	53.3 (9.4)	−2.47 (15.72)	[−7.08, 2.15]	−1.1	.286	0.15
Social Potency	54.6 (8.3)	60.1 (7.3)	−5.65 (11.21)	[−8.94, −2.36]	−3.4	.001*	0.49
Achievement	56.3 (7.1)	57.2 (8.8)	−0.82 (10.94)	[−4.03, 2.39]	−0.6	.529	0.09
Social Closeness	53.0 (7.7)	56.3 (7.3)	−3.24 (10.76)	[−6.40, −0.08]	−2.1	.041	0.31
Negative Emotionality	51.8 (8.1)	52.6 (7.7)	−0.85 (11.69)	[−4.28, 2.58]	−0.5	.653	0.07
Stress Reaction	55.7 (9.7)	54.0 (9.4)	1.79 (15.01)	[−2.62, 6.19]	0.8	.452	0.11
Aggression	44.9 (4.3)	44.5 (4.7)	0.26 (5.77)	[−1.43, 1.95]	0.4	.675	0.06
Alienation	52.9 (9.5)	55.4 (10.7)	−2.77 (14.22)	[−6.94, 1.41]	−1.2	.238	0.17
Constraint	45.6 (8.1)	42.7 (9.0)	3.02 (11.26)	[−0.28, 6.33]	1.7	.092	0.25
Control	53.6 (8.9)	47.4 (10.9)	6.27 (13.92)	[2.18, 10.36]	3.0	.004*	0.43
Harm Avoidance	49.5 (8.6)	48.3 (9.6)	1.22 (11.21)	[−2.07, 4.51]	0.7	.483	0.10
Traditionalism	36.7 (7.8)	38.9 (7.6)	−2.07 (11.92)	[−5.57, 1.43]	−1.3	.203	0.19
Absorption	61.0 (8.4)	60.8 (9.1)	0.41 (12.13)	[−3.14, 3.98]	0.1	.910	0.02

*Note.* Comparisons with * indicate significance at a family-wise alpha correction (Positive Emotionality trait family α = .01; Negative Emotionality and Constraint trait families α = .0125; Absorption α = .05).

A stepwise logistic regression selected two trait facets from the 11 facets entered into the model to classify the data into singers with nodules and singers with healthy voices: Social Potency (β weight = .077, *OR* = 1.080, *p* = .010) and Control (β weight = −.051, *OR* = 0.950, *p* = .036). These results indicate that with an increase in 1 point on the Social Potency score, the odds of the individual being a patient increases by 8%; for each point decrease in Control, the odds of being a patient increases by 5%. Because the scores are standardized *T* scores, for each *SD* (i.e., 10 points) increase in Social Potency, the chances of being a patient increase by 80%; for every *SD* decrease in Control, the chances of being a patient increase by 50%. The resulting overall classification of the model was 69.1% accurate (true positives = 33, true negatives = 32, false positives = 15, false negatives = 14, AUC = .719). A separate stepwise logistic regression model, which used the three higher order traits, selected one trait to discriminate between groups: PEM (β weight = .064, *OR* = 1.066, *p* = .011). For each point increase in PEM, the odds of being a patient increase by 6.6%. This model showed an overall classification accuracy of 64.9% (true positives = 32, true negatives = 29, false positives = 18, false negatives = 15, AUC = .681).

Table 3 shows results for the Pearson correlation coefficients for the pairwise analyses between higher order personality traits (i.e., PEM, NEM, and CON) and weekly speaking voice parameters for each of the two groups of singers (i.e., those with healthy voices and those with nodules). The pairwise analyses between personality facets and speaking voice parameters for singers with nodules are presented in Table 4 and for healthy singers in Table 5. Since this study aim is exploratory, we attempted to reduce our risk of Type II errors (i.e., identifying no effect when a true effect exists) by presenting the strength of the correlations and raw significance values.

**Table 3.** Pearson *r* correlation coefficients for the relationship between the higher-order traits of Positive Emotionality (PEM), Negative Emotionality (NEM), and Constraint (CON), and weeklong speaking voice use distributional parameters, presented separately for singers with healthy voices and singers with phonotrauma.

Ambulatory voice parameter	Vocally healthy singers			Singers with nodules		
	PEM	NEM	CON	PEM	NEM	CON
Dose						
Phonation time (%)	.38**	.14	.02	.17	−.10	.04
Cycle (cycles/hr)	.36*	.16	−.02	.18	−.04	−.03
Distance (m/hr)	.36*	−.05	−.18	.24	.03	.04
SPL						
*M* (dB SPL)	.25	−.24	−.28	.21	.08	.07
*SD* (dB SPL)	.18	.16	−.39**	−.04	.08	−.12
Minimum (dB SPL)	.11	−.39**	.01	.16	.04	.15
Maximum (dB SPL)	.28	−.08	−.39**	.13	.12	.00
Range (dB SPL)	.21	.16	−.39**	−.01	.08	−.11
Skewness	.08	−.03	−.04	.03	.00	.04
Kurtosis	−.07	−.22	.30*	−.19	.15	.05
NSAM level						
Skewness	−.16	.23	.10	−.31*	.17	−.06
Kurtosis	.03	−.13	.22	.04	−.05	.15
*f_o_*						
Mode (Hz)	.13	.06	−.27	.18	.14	−.25
*SD* (Hz)	−.18	−.27	.06	.10	.12	.01
Minimum (Hz)	.08	.17	−.34*	.17	.05	−.25
Maximum (Hz)	−.12	−.18	−.10	.08	.12	−.10
Range (Hz)	−.19	−.31*	.07	.02	.12	.01
Skewness	−.10	.09	.12	.20	−.09	.15
Kurtosis	−.04	.22	.07	.17	−.05	.16
CPP						
*M* (dB)	.42**	−.06	−.13	.03	−.02	.05
*SD* (dB)	.37*	−.19	−.05	−.09	.02	−.03
Minimum (dB)	.38**	.04	−.19	.11	−.07	.10
Maximum (dB)	.41**	−.10	−.12	−.04	−.01	.05
Range (dB)	.38**	−.17	−.06	−.11	.03	.00
Skewness	−.35*	−.01	.07	−.12	−.01	.02
Kurtosis	.05	.17	−.09	.04	.11	.10
*H*_1_ *–H*_2_						
*M* (dB)	−.31*	−.01	.22	−.35*	.11	.15
*SD* (dB)	.03	−.21	.00	−.01	.20	−.11
Minimum (dB)	−.25	.05	.19	−.30*	−.01	.14
Maximum (dB)	−.28	−.07	.24	−.35*	.18	.09
Range (dB)	−.02	−.15	.04	−.03	.22	−.08
Skewness	.14	.14	−.01	.02	−.11	−.04
Kurtosis	.18	.16	−.06	.18	−.26	−.06

*Note.* SPL = sound pressure level; NSAM = neck surface acceleration magnitude; *f_o_* = fundamental frequency; CPP = cepstral peak prominence; *H*_1_ *–H*_2_ = the difference between the first two harmonic magnitudes.

* *p* ≤ .05.

** *p* ≤ .01.

**Table 4.** Pearson *r* correlation coefficients for the relationship between lower-order trait facets and weeklong speaking voice use distributional parameters in singers with phonotrauma.

Ambulatory voice parameter	Wellbeing	Social Potency	Achievement	Social Closeness	Stress Reaction	Aggression	Alienation	Control	Harm Avoidance	Traditionalism	Absorption
Dose											
Phonation time (%)	.11	.13	.22	.05	−.16	−.04	−.02	.03	−.05	.13	.21
Cycle (cycles/hr)	.13	.14	.27	.00	−.16	−.03	.07	.02	−.15	.11	.25
Distance (m/hr)	.19	.17	.26	.08	−.16	.03	.14	.11	−.23	.27	.13
SPL											
*M* (dB SPL)	.15	.22	.11	.14	−.04	.06	.08	.14	−.21	.28	−.13
*SD* (dB SPL)	−.03	−.13	.14	−.11	−.07	.03	.18	.02	−.24	−.01	.00
Minimum (dB SPL)	.14	.21	−.02	.13	.05	.07	−.05	.11	.00	.21	−.08
Maximum (dB SPL)	.11	.06	.15	.02	−.02	.10	.15	.14	−.26	.17	−.07
Range (dB SPL)	−.01	−.11	.15	−.08	−.06	.03	.17	.04	−.23	−.01	.00
Skewness	.18	−.10	.05	−.04	.09	.13	−.11	.12	.03	−.15	.01
Kurtosis	−.24	−.03	−.19	−.13	.17	.07	.08	−.07	.18	.01	.13
NSAM level											
Skewness	−.21	−.39**	−.23	−.13	.01	.18	.24	−.01	.04	−.18	.24
Kurtosis	−.03	.27	−.07	.00	.20	−.06	−.24	.16	.00	.12	−.09
*f_o_*											
Mode (Hz)	.22	.16	.19	−.07	−.09	.18	.21	−.16	−.22	−.10	.20
*SD* (Hz)	.08	.07	.20	−.09	.14	.01	.06	.05	−.05	.01	.05
Minimum (Hz)	.17	.12	.22	−.04	−.16	−.04	.23	−.11	−.28	−.06	.20
Maximum (Hz)	.07	.06	.23	−.15	.06	−.02	.15	.00	−.16	−.04	.11
Range (Hz)	.00	.01	.18	−.17	.16	.00	.07	.06	−.06	−.02	.04
Skewness	.24	.05	.10	.22	−.04	.03	−.16	.03	.15	.12	−.04
Kurtosis	.22	−.02	.10	.22	−.03	.14	−.14	.07	.13	.10	.04
CPP											
*M* (dB)	−.04	.08	−.02	.10	.04	.14	−.13	−.05	.09	.06	−.12
*SD* (dB)	−.13	.01	−.13	−.02	−.03	−.03	.06	−.18	.12	.06	−.08
Minimum (dB)	.05	.07	.08	.15	.01	.23	−.23	.04	.10	.02	−.12
Maximum (dB)	−.10	.04	−.09	.03	.01	.09	−.06	−.11	.15	.07	−.10
Range (dB)	−.15	.01	−.16	−.04	.00	−.01	.05	−.16	.14	.08	−.06
Skewness	−.04	−.14	−.05	−.15	−.11	−.14	.14	−.03	.09	−.01	.07
Kurtosis	−.01	.16	.07	−.08	.23	.13	−.08	.22	−.12	.05	−.02
*H*_1_ *–H*_2_											
*M* (dB)	−.21	−.26	−.28	−.34*	.15	−.08	.13	.23	.12	−.10	.15
*SD* (dB)	−.03	−.03	.06	−.09	.06	−.05	.28	−.08	−.04	−.08	.20
Minimum (dB)	−.17	−.21	−.27	−.25	.10	−.06	−.03	.20	.08	−.04	.02
Maximum (dB)	−.22	−.25	−.25	−.36*	.19	−.11	.22	.19	.06	−.11	.21
Range (dB)	−.05	−.03	.04	−.10	.09	−.05	.28	−.04	−.03	−.08	.21
Skewness	.00	.12	−.05	.04	.09	.00	−.25	.04	−.17	.06	−.21
Kurtosis	.06	.17	.14	.27	−.09	.08	−.38**	−.01	−.16	.02	−.31*

*Note.* SPL = sound pressure level; NSAM = neck surface acceleration magnitude; *f_o_* = fundamental frequency; CPP = cepstral peak prominence; *H*_1_ *–H*_2_ = the difference between the first two harmonic magnitudes.

* *p* ≤ .05.

** *p* ≤ .01.

**Table 5.** Pearson *r* correlation coefficients for the relationship between lower-order trait facets and weeklong speaking voice use distributional parameters in singers with healthy voices.

Ambulatory voice parameter	Wellbeing	Social Potency	Achievement	Social Closeness	Stress Reaction	Aggression	Alienation	Control	Harm Avoidance	Traditionalism	Absorption
Dose											
Phonation time (%)	.34*	.30*	−.04	.26	−.04	.24	.12	−.18	.07	.22	−.18
Cycle (cycles/hr)	.32*	.30*	−.08	.26	−.06	.21	.12	−.20	.06	.18	−.21
Distance (m/hr)	.38**	.34*	−.15	.25	−.19	.28	−.08	−.28	−.12	.09	−.31*
SPL											
*M* (dB SPL)	.37*	.21	−.12	.14	−.26	.09	−.24	−.20	−.26	−.08	−.31*
*SD* (dB SPL)	.14	.29*	.05	−.07	.12	.27	.07	−.17	−.44**	−.17	.03
Minimum (dB SPL)	.27	.01	−.18	.17	−.38**	−.07	−.34*	−.09	.08	.03	−.40**
Maximum (dB SPL)	.33*	.33*	−.05	.05	−.12	.21	−.14	−.23	−.38**	−.16	−.20
Range (dB SPL)	.16	.31*	.06	−.06	.12	.25	.08	−.17	−.43**	−.18	.05
Skewness	.03	.24	−.09	.01	.00	.06	−.09	−.07	.11	−.16	−.08
Kurtosis	.01	−.21	.06	.02	−.19	−.36*	−.06	.19	.34*	.05	−.12
NSAM level											
Skewness	−.29	−.01	.05	−.13	.27	.13	.12	.13	.06	−.03	.17
Kurtosis	.10	−.20	.32*	−.08	−.13	−.21	−.01	.19	.16	.05	−.12
*f_o_*											
Mode (Hz)	.04	.23	−.19	.18	.05	.00	.06	−.22	−.13	−.17	−.04
*SD* (Hz)	.01	−.16	−.30*	−.01	−.21	−.18	−.19	.07	.04	.01	−.25
Minimum (Hz)	.01	.21	−.07	.00	.17	−.03	.17	−.19	−.21	−.28	.06
Maximum (Hz)	.02	−.02	−.28	−.04	−.13	−.20	−.10	−.04	−.03	−.11	−.16
Range (Hz)	.02	−.14	−.30*	−.05	−.25	−.22	−.21	.06	.08	.02	−.23
Skewness	−.08	−.18	−.01	.02	.17	−.04	.03	.22	.04	−.07	−.03
Kurtosis	−.04	−.16	.05	.03	.23	.03	.18	.13	.02	−.04	.06
CPP											
*M* (dB)	.36*	.28	.10	.27	−.29	.27	.01	−.21	−.17	.18	−.33*
*SD* (dB)	.38**	.18	.09	.26	−.29*	.15	−.18	−.09	−.26	.33*	−.17
Minimum (dB)	.29*	.28	.05	.26	−.22	.33*	.10	−.26	−.13	.07	−.37*
Maximum (dB)	.38**	.25	.08	.29	−.29*	.26	−.06	−.18	−.23	.24	−.29
Range (dB)	.38**	.19	.09	.26	−.29*	.18	−.15	−.10	−.26	.32*	−.19
Skewness	−.28	−.26	−.14	−.17	.23	−.16	−.13	.18	.00	−.09	.33*
Kurtosis	−.05	.13	.16	−.09	.05	.04	.25	−.07	.14	−.30*	−.02
*H*_1_ *–H*_2_											
*M* (dB)	−.14	−.30*	.03	−.34*	.09	−.17	.01	.22	.05	.16	.33*
*SD* (dB)	.04	.01	−.03	.08	−.10	−.11	−.23	.20	−.23	.02	.03
Minimum (dB)	−.12	−.22	.03	−.30*	.10	−.16	.08	.11	.15	.14	.30*
Maximum (dB)	−.16	−.24	.03	−.29*	.06	−.25	−.05	.33*	−.02	.16	.34*
Range (dB)	−.04	−.01	−.01	.03	−.05	−.09	−.16	.24	−.20	.02	.02
Skewness	.01	.24	.04	.03	.06	−.06	.19	−.08	.16	−.10	−.03
Kurtosis	−.02	.20	.03	.22	−.03	.20	.21	−.25	.20	−.07	−.30*

*Note.* SPL = sound pressure level; NSAM = neck surface acceleration magnitude; *f_o_* = fundamental frequency; CPP = cepstral peak prominence; *H*_1_ *–H*_2_ = the difference between the first two harmonic magnitudes.

* *p* ≤ .05.

** *p* ≤ .01.

The intent of the multiple regression analyses was to identify the relative contributions of certain personality traits/facets for each voice outcome measure that had more than one associated personality trait. No multiple regression analyses were conducted for the higher order traits because none of the voice outcome measures were associated with more than one higher-order trait. Notably, the singers with nodules only had one voice parameter (*H*_1_ *–H*_2_ kurtosis) that was associated with more than one trait facet (Alienation and Absorption). The overall model of the multiple regression conducted with these variables was significant, *F*(2, 44) = 4.62, *p* = .015, *R* = .42. The model *R* ^2^ was .17, indicating that Alienation and Absorption facets accounted for 17% of the variance in the weeklong speaking distribution of *H*_1_ *–H*_2_ kurtosis. Alienation was a larger contributing factor to the model (β = −.31, *p* = .045, partial *r* ^2^ = .08) than Absorption (β = −.18, *p* = .245, partial *r* ^2^ = .03).

Several multiple regression models were conducted on the group of singers with healthy voices because several voice outcome measures correlated with more than one trait facet. These regression model statistics are presented in Table 6. Each multiple regression model in this group was statistically significant, with the overall multiple *R* ranging from .39 to .58. Vocal dose measures were positively associated with a combination of Wellbeing and Social Potency facets, with the addition of a negative correlation between Absorption and distance dose. Mean SPL was positively correlated with Wellbeing and negatively correlated with Absorption. SPL variability and range measures were positively correlated with Social Potency and negatively correlated with Harm Avoidance. None of the *f_o_* measures had more than one significantly correlated facet; therefore, no multiple regression models were conducted. In fact, the only significant pairwise correlations among *f_o_* distributional parameters and facets were between Achievement and *f_o_* *SD* and range, with higher Achievement scores being associated with smaller pitch-related variability and range. CPP distributional parameters were associated with several PEM-related facets. Wellbeing was the only and consistently the strongest facet that was associated with each of the CPP distributional parameters. Higher Wellbeing scores were associated with larger CPP variability and a tendency for higher weeklong CPP distributional values (i.e., minimum, mean, and maximum CPP values tend to be higher), even when controlling for other associated facets. Lower *H*_1_ *–H*_2_ parameters tended to be associated with higher social inclinations, as the combination of Social Closeness, Social Potency, and Absorption was associated with mean *H*_1_ *–H*_2_. Interestingly, Absorption was a significant variable in the model even when controlling for Social Potency and Social Closeness.

**Table 6.** Multiple regression model statistics completed on the cohort of singers with healthy voices (*n* = 47) using weeklong distributional measures of speaking voice use as the dependent variable and personality facets as the independent variables.

Dependent variable	Model *R* ^2^	Independent variables	β	*t*	*p*	Partial *r* ^2^
Phonation time	.15*					
		Wellbeing	.265	1.79	.081	.06
		Social Potency	.206	1.39	.172	.04
Cycle dose	.15*					
		Wellbeing	.248	1.68	.101	.05
		Social Potency	.217	1.46	.150	.04
Distance dose	.29**					
		Wellbeing	.310	2.26	.029	.09
		Social Potency	.221	1.61	.114	.04
		Absorption	−.313	−2.43	.019	.10
SPL mean	.24**					
		Wellbeing	.375	2.84	.007	.14
		Absorption	−.320	−2.43	.019	.10
SPL *SD*	.24**					
		Social Potency	.210	1.56	.125	.04
		Harm Avoidance	−.401	−2.99	.005	.16
SPL minimum	.26**					
		Stress Reaction	−.171	−1.09	.280	.02
		Alienation	−.224	−1.55	.129	.04
		Absorption	−.291	−2.03	.049	.07
SPL maximum	.23**					
		Wellbeing	.170	1.14	.260	.02
		Social Potency	.211	1.48	.147	.04
		Harm Avoidance	−.279	−1.96	.057	.07
SPL range	.24**					
		Social Potency	.239	1.78	.083	.06
		Harm Avoidance	−.379	−2.89	.007	.14
SPL kurtosis	.18*					
		Aggression	−.279	−1.91	.063	.07
		Harm Avoidance	.239	1.64	.109	.05
CPP mean	.24**					
		Wellbeing	.366	2.79	.008	.13
		Absorption	−.341	−2.60	.013	.12
CPP *SD*	.23**					
		Wellbeing	.308	2.12	.040	.08
		Stress Reaction	−.090	−0.59	.556	.01
		Traditionalism	.261	1.85	.072	.06
CPP minimum	.33**					
		Wellbeing	.309	2.47	.017	.10
		Aggression	.320	2.56	.014	.10
		Absorption	−.367	−2.94	.005	.13
CPP maximum	.17*					
		Wellbeing	.313	2.09	.042	.08
		Stress Reaction	−.168	−1.13	.267	.02
CPP range	.22*					
		Wellbeing	.305	2.09	.042	.08
		Stress Reaction	−.093	−0.61	.546	.01
		Traditionalism	.248	1.75	.087	.06
*H*_1_ *–H*_2_ mean	.23*					
		Social Potency	−.217	−1.50	.140	.04
		Social Closeness	−.193	−1.32	.192	.03
		Absorption	.284	2.07	.045	.08
*H*_1_ *–H*_2_ minimum	.15*					
		Social Closeness	−.249	−1.75	.086	.06
		Absorption	.249	1.75	.086	.06
*H*_1_ *–H*_2_ minimum	.14*					
		Social Closeness	−.200	−1.34	.189	.04
		Control	.254	1.70	.097	.06

*Note.* SPL = sound pressure level; CPP = cepstral peak prominence; *H*_1_ *–H*_2_ = the difference between the first two harmonic magnitudes.

* *p* ≤ .05.

** *p* ≤ .01.

Appendix B shows group-based descriptive statistics for all ambulatory speaking voice parameters. These results indicate that, comparable to our previous findings (Toles et al., 2021), singers with nodules have higher vocal doses in speech, higher average and more negatively skewed SPL distributions, and more restricted *H*_1_ *–H*_2_ distributions than vocally healthy singers.

## Discussion

### Group Differences in Personality Traits

This work sought to determine if certain personality traits are significantly related to the presence of vocal fold nodules in singers and/or to the types of daily voice use that could contribute to the etiology and pathophysiology of phonotrauma in singers. Initial studies testing the TTVD found that female individuals with vocal fold nodules scored higher on Social Potency and lower on Control facets than individuals with healthy voices, but these groups were composed mainly of nonsingers (Roy et al., 2000a). Until this study, there was an absence of research investigating whether the TTVD is similarly relevant to singers (i.e., Do certain personality traits in singers indicate an increased risk for developing vocal fold nodules?). Furthermore, the personality traits of singers have only been described in a handful of studies, most of which included a relatively small number of singers (with undisclosed vocal health status) among a larger group composed of different types of musicians (Butkovic et al., 2015; Heller et al., 2015; Kemp, 1981, 1996; Miksza, 2006). Like those studies, this study found that healthy singers, along with singers with nodules, had higher levels of extraversion-related traits (i.e., PEM and its facets). Singers with healthy voices scored approximately half of an *SD*, on average, higher than the population mean (Patrick et al., 2002) on PEM and the facets of Social Potency and Achievement. This finding suggests that singers with healthy voices tend to be extraverted, socially dominant, and high achievers compared to the average population.

In this study, the singers with nodules tended to have even higher levels on all extraversion-related traits compared to the healthy matched-control singers. Though Social Potency was the only extraversion-related trait scale that was significantly different between the groups at the corrected significance value, the PEM and Social Closeness scales both trended higher in the nodules group with small-to-medium effect sizes. We applied a statistical correction to our significance value, which resulted in these two scales not meeting the conservative level of significance. However, both scales were within the traditional, uncorrected significance levels. The 95% confidence intervals presented in Table 2 support the idea that these scales are representative of differences between groups and suggest that, with increased power, those results might become statistically significant at the corrected value. Singers with nodules scored greater than one full *SD* above the population mean on PEM and greater than one half of an *SD* on Social Closeness. This is an important finding considering that singers with nodules also had significantly lower scores on the Control facet, reflecting higher levels of impulsivity, compared with their healthy singer counterparts. The tendency to seek out social situations (i.e., higher scores on Social Closeness) combined with significantly higher levels of social dominance (i.e., higher scores on Social Potency) within the context of elevated impulsivity (i.e., lower scores on Control) could potentially lead to more frequent scenarios in which the singer with nodules might be more likely to speak more often and more loudly (with increased laryngeal forces), which could increase risk and/or perpetuation of phonotrauma. It is noteworthy that the stepwise discriminant analysis, which used the Social Potency and Control facets as the discriminant factors, was relatively accurate (69.1%) using personality trait facets alone. These results imply that scores on two personality scales can discriminate between a healthy singer and a singer with nodules greater than two thirds of the time, without even considering other factors that might be more challenging to measure (e.g., daily voice use patterns). Furthermore, the same two facet scales were identified as discriminatory characteristics between female individuals with nodules and vocally healthy controls, who were not necessarily singers, in a past study (Roy et al., 2000a). These results support the notion that an individual's proclivity for social dominance combined with impulsivity can increase their risk for developing phonotrauma. These results also suggest that screening young singers using a personality questionnaire could potentially identify those who are at higher risk for having and/or developing phonotrauma.

One of the surprising findings in this study was that there were no differences between groups on the NEM scale (i.e., related to the concept of Neuroticism) or its underlying facet scales. We hypothesized that singers with nodules would exhibit higher scores on these traits based on previous findings using nonsingers, in which individuals with nodules had higher levels of neuroticism-related traits compared to healthy controls (Roy et al., 2000a, 2000b). Although the NEM-related traits/facets showed no differences between groups in this study, both the singers with nodules and the singers with healthy voices exhibited higher-than-average levels of Stress Reaction. Stress Reaction is a trait facet that has been linked with heightened autonomic nervous system response (i.e., fight or flight; Ziegler, 2012) and has been associated with increased intrinsic laryngeal muscle activation (Helou et al., 2013, 2018, 2020). Such laryngeal muscle activation could hypothetically lead to development or persistence of vocal hyperfunction patterns. High neuroticism-related traits, such as Stress Reaction, are theorized to increase the propensity for either extroverted or introverted behavior (Eysenck & Levey, 1972; Matthews & Gilliland, 1999). For extraverts (i.e., both groups of singers in this study), this would theoretically increase their inclination to exhibit active/approach behaviors (Gray, 1981), which could manifest vocally as frequent and/or loud speech, commonly associated with increased risk of phonotrauma.

The one MPQbf trait scale that is not related to any other scale is Absorption, which can be described as the inclination to engage in imaginative or self-involved experiences (Patrick et al., 2002). There were not differences between groups on this scale, but both groups of singers presented with substantially high scores (greater than one full *SD* above the population mean). This indicates that singers, regardless of their vocal status, have more tendency to be self-engaged, imaginative, and responsive to engaging stimuli. High levels of Absorption could have implications for how singers use their voices, such as being more vocally responsive to a social setting.

Whereas both groups of singers tended to score high on the Absorption scale and the traits related to Extraversion (specifically those related to social dispositions) and Neuroticism (the trait of Stress Reaction), it is important to note that singers with nodules integrated those baseline characteristics with significantly higher levels of Social Potency and significantly lower levels of Control. This blend of traits uniquely discriminated singers with nodules from healthy singers, and when confronted with the opportunity to occupy the social limelight (i.e., elevated Social Potency), such singers may be less able to limit vocal behavior leading to phonotrauma despite audible signs of voice deterioration (i.e., reflecting lower control). However, the trait profiles of both groups suggest that singers, in general, might be at higher risk for employing phonotraumatic patterns in their daily speaking voice use.

### Relationships Between Personality and Daily Voice Use

Since personality is theorized to influence behavior (Twenge & Campbell, 2016), we hypothesized that singers would display specific relationships between personality traits and speaking voice use. We decided to exclude singing from the analysis because (a) our theory posits that personality would primarily influence speaking voice use in singers (e.g., speaking more often) due to their higher levels of socially inclined extraversion-related traits, which would contribute to increased risk of phonotrauma when combined with their singing vocal demands, and (b) our recent study found that daily singing voice use was not significantly different between singers with phonotrauma and vocally healthy singers (Toles et al., 2021).

This study found several relationships between speaking voice use and personality traits and facets, findings that have implications for individualizing efforts related to the prevention, assessment, and treatment of PVH in singers. The observed relationships account for up to 33% of the variance in some single parameters of speaking voice, which is substantial considering that these are innate personal characteristics. These results provide further evidence for the notion that daily voice use is, at least secondarily, influenced by psychosocial-related factors.

### Vocally Healthy Singers

Nearly all the relationships between speaking voice use and personality were found within the control group of singers with healthy voices as opposed to the singers with nodules, which is a broad finding that we did not anticipate. Though it was unexpected that correlations occurred mainly in vocally healthy singers, most of these relationships were associated with medium-to-strong effect sizes (J. Cohen, 1988) and were consistent with our theory about how personality might influence speaking voice use. Some might consider the correlations described in this investigation to be relatively small, considering other studies in speech research (Gaeta & Brydges, 2020). We based our interpretation of the correlation effect sizes on Cohen's interpretation for social sciences (J. Cohen, 1988), which indicates that correlations of .30 are medium and correlations of .50 are large. Within the context of psychology research and personality research, specifically, correlations tend to be smaller than in other disciplines because of the notion that human psychology is intrinsically complex and difficult to measure. In fact, recent literature has suggested interpretations of .10 as small, .20 as medium, .30 as large, and .40 or greater as very large in psychological research (Funder & Ozer, 2019). Viewed in this context, we consider the relationships between personality traits and voice measures described in this study to be genuine. Furthermore, this study aim was exploratory, and we hope to use these findings to develop future, more specific hypotheses. Therefore, we made the decision to avoid the conservative step of correcting the alpha level of significance for the primary reason of reducing the risk of Type II errors. The correlations illustrate a clear pattern that is clinically and theoretically meaningful, which alleviates concern about spurious results.

As hypothesized, PEM and its related facets were associated with speaking vocal dose. Healthy singers with a lower threshold for experiencing positive emotions, specifically those associated with happiness (i.e., Wellbeing) and leadership/assertiveness (i.e., Social Potency), tended to have increased speaking voice use. Higher Wellbeing, along with lower Absorption, was also associated with higher mean SPL, implying that happier individuals who are less self-involved will speak louder on average. Therefore, we theorize that singers who are more dominant and happier might be at an increased risk for using more intense speaking voice use patterns that could predispose them to develop phonotrauma.

Lower CON scores were associated with increased SPL variability that was mostly accounted for by an increase in the upper end of the range. We expected the Control facet to be the primary force in these relationships since it reflects impulsivity and is discriminative between patients and controls, but investigation into the facets showed that Harm Avoidance was the strongest CON-related variable associated with SPL range/variability, even when controlling for other correlated facets. It is reasonable to expect low Harm Avoidance to be a factor in the SPL distribution because the scale represents the tendency to avoid risk of injury; therefore, those singers who are less responsive to threat of injury (and likely less cautious) have the tendency to employ a wider range of vocal intensity, specifically toward the higher end of the SPL range (as evidenced by the negative correlation with maximum SPL values and lack of correlation with minimum SPL values). Even though the relationship between Harm Avoidance and SPL range was already medium to strong (*r* = −.43), the addition of Social Potency resulted in an even stronger model (*R* = .49). The combination of low Harm Avoidance, high Social Potency, and high Wellbeing was related to higher maximum SPL values (*R* = .48). These results suggest that socially dominant individuals who take less caution to evade injury are more likely to engage in a wider intensity range, specifically by extending the higher (louder) end of the range.

PEM has a medium-to-strong relationship with every CPP distributional parameter, except kurtosis, in the healthy singer group. Again, Wellbeing was found to be the facet that was the most dominant in these relationships. Higher levels of Wellbeing were associated with higher average CPP, increased CPP range and variability, as well as higher maximum and higher minimum CPP values. This suggests that individuals who are happier tend to speak with increased periodic energy, which is a novel finding. We attempted to account for the possibility that SPL and/or *f_o_* might have an indirect effect on the relationship between the personality traits and measures of CPP since those features have previously been shown to influence CPP (Brockmann-Bauser et al., 2019). A separate mediation analysis was conducted for each of the significant correlations between personality and CPP measures with SPL and *f_o_* included as mediation variables. There were no indirect mediation effects for SPL or *f_o_* identified in any of the models, which could indicate that the relationships between personality and CPP parameters are not due to these parameters.

*H*_1_ *–H*_2_ is a measure that has been associated with the level of glottal closure (Klatt & Klatt, 1990) and can be confidently estimated from the accelerometer signal (Mehta et al., 2019). Lower *H*_1_ *–H*_2_ values indicate increased/pressed glottal closure (Klatt & Klatt, 1990). One recent study found that singers with nodules speak with lower average *H*_1_ *–H*_2_ throughout the course of a week and with significantly reduced variability in the weeklong distribution, suggesting that they speak with more abrupt and pressed glottal closure compared to vocally healthy matched-controls (Toles et al., 2021). Two additional recent studies provide further evidence to this effect (Van Stan, Mehta, Ortiz, Burns, Marks, Toles, Stadelman-Cohen, et al., 2020; Van Stan, Mehta, Ortiz, Burns, Marks, Toles, Vangel, et al., 2020). Based on the findings from these recent studies, lower and more restricted *H*_1_ *–H*_2_ distributions were theorized to increase an individual's risk for developing phonotrauma. Therefore, it is interesting that vocally healthy singers show negative relationships between mean *H*_1_ *–H*_2_ and Social Potency (which is significantly higher in the nodules group) and Social Closeness (which tends to be higher in the nodules group). Negative relationships were also found between Social Closeness and the minimum and maximum *H*_1_ *–H*_2_ values, suggesting that distributions of *H*_1_ *–H*_2_ tend to be shifted lower (i.e., increased glottal closure) for singers who are more socially inclined. These finding suggest that perhaps singers without current pathology who possess higher levels of sociality and social dominance are more at risk for engaging in speaking voice use that tends to be associated with phonotrauma, theoretically increasing their risk for developing phonotrauma in the future.

### Singers With Nodules

One of the most surprising findings in this study was that singers with nodules displayed either weak or nonexistent relationships between most personality traits and daily use of speaking voice. Notably, however, the only relationships found were among voice variables previously shown to discriminate patients with phonotrauma from matched control participants (Toles et al., 2021; Van Stan, Mehta, Ortiz, Burns, Marks, Toles, Stadelman-Cohen, et al., 2020; Van Stan, Mehta, Ortiz, Burns, Marks, Toles, Vangel, et al., 2020): skewness of the NSAM distribution and mean and variability of the *H*_1_ *–H*_2_ distribution. These past studies indicated that singers with nodules already demonstrate lower *H*_1_ *–H*_2_ values and negatively skewed NSAM distributions, presumably reflecting compensation for the presence of vocal fold pathology. The results of this study suggest that these vocal features are even more pronounced in singers with nodules who have higher levels of Social Potency. This could be attributed to the fact that singers with these traits tend to engage more in social activities and when combined with their need to compensate for the presence of laryngeal pathology results of the use of higher laryngeal forces and hyperadduction of the vocal folds to attain a functional voice quality more often than singers with lower levels of these traits.

As shown in Appendix B, the voice use differences between singers with nodules and vocally healthy singers included in this study align with our previous report (i.e.,singers with nodules speak with higher vocal doses than vocally healthy singers; Toles et al., 2021). It is puzzling that even though singers with nodules speak more than vocally healthy singers, they show no relationships between personality and vocal dose in speech. One explanation is that the nodules group shows somewhat of a ceiling effect in their personality scores, particularly in PEM and PEM-related facets. There is moderate-to-large negative skewness in the PEM trait and facet scales in this group, whereas the group of vocally healthy singers had relatively normal distributions of scores. This reduction of range at the higher end of the scale likely attenuates the strength of relationships. Furthermore, we expect that overall vocal function is likely limited in this group due to the presence of vocal fold pathology, which could be obscuring potential associations between personality and types of voice use in speech that could have been present prior to development of lesions.

Another explanation for the paucity of relationships in the nodules group could be attributed to the Hawthorne effect, which is a phenomenon that suggests that people change their behavior when they know they are being observed (McCambridge et al., 2014). Both groups were monitored in their daily lives using a voice monitor that could potentially serve as a frequent reminder that they are being observed and could result in atypical vocal behavior. However, this effect may be even more relevant to singers who have been recently diagnosed with nodules who may change both their singing and speaking voice use after discussing the etiology and management of the nodules with their clinicians. Therefore, it is possible that these data are actually an underrepresentation of speaking voice use in singers with nodules. Even though they speak significantly more than vocally healthy singers, their unobserved speaking voice use might be substantially different (e.g., higher vocal dose) and thus more strongly related to personality traits.

### Limitations and Future Directions

The findings from this study indicate that vocally healthy singers do in fact display a relationship between personality traits and speaking voice use, which could be consequential in how we might frame our perspectives on the prevention of phonotrauma. Many of the singers in this study were relatively early in their careers, and possessing certain personality traits could potentially represent a significant risk that may be more important over time, as their careers progress and vocal demands increase. Therefore, even young singers with no current pathology might carry a higher risk for developing phonotrauma in the future based on their personality traits. A limitation to this study design is that, although ambulatory data were collected in participants' real-world settings, it is still considered an isolated snapshot in time and does not provide information as to what happens over time for these individuals. A longitudinal study investigating the relationships between personality and changes in vocal status over time might shed light on the effect of certain personality traits on daily voice use and subsequent development of phonotrauma in this high-risk population, ultimately improving early identification of individuals at increased risk. Efforts to educate and counsel young, at-risk singers about how their personality profiles could predispose them to damaging patterns of voice use could potentially mitigate their risk of developing phonotrauma.

In addition to the increased risk singers have of developing phonotrauma, individuals who have been diagnosed with phonotrauma are more likely to have recurrent voice problems (Hillman et al., 2020; Lee et al., 2021). This is often attributed to the notion that a past injury to vocal fold tissue might predispose the tissue to repetitive injury, but it is possible that a contributing factor to the phonotraumatic cycle includes durable personality traits related to extraversion and impulsivity. It is logical that an individual who enjoys social activities, is assertive, and does not avoid risk of injury would have difficulty changing (in a long-term, sustained manner) the damaging patterns of voice use associated with those enduring personality traits. Perpetuation of these patterns could contribute to relapses. This study is insufficient to make such claims. Future work should investigate long-term implications of personality traits on vocal status following initial diagnosis of vocal fold nodules or other phonotraumatic pathology, specifically as it relates to adherence to voice therapy and/or medical recommendations.

The MPQbf scale was chosen based on the idea that it would best measure our conceptual framework. However, it only measures three higher-order traits. Scales that measure the “big five” higher-order traits and their facets, such as the NEO Personality Inventory (Costa & McCrae, 1992), could provide an additional level of granularity. Specifically, one higher-order trait that would be interesting to assess in singers with nodules is Agreeableness, which has implications for adherence to voice recommendations. Individuals high in Agreeableness are more cooperative, trusting, and sympathetic (Twenge & Campbell, 2016), which could theoretically affect the relationship they have with their clinician and their motivation to comply with medical and/or therapeutic recommendations.

Although this study is the first to identify relationships between personality and voice use in the daily life of singers, the specific relationships identified here might not be generalizable to the general (nonsinger) population. As we reported in the first stage of analysis, even healthy singers tended to have higher-than-average levels of PEM and its related facets as well as higher-than-average levels of Stress Reaction, a finding that might reduce the potential to find relationships between personality and voice use in healthy nonsingers. Furthermore, the dysphonic population in this study included only singers with phonotrauma, but many singers can also experience NPVH (i.e., muscle tension dysphonia). We cannot rule out that singers with NPVH will have an entirely different personality profile (e.g., lower PEM and higher NEM) than singers with PVH or even healthy singers, and we expect singers with NPVH to have different daily speaking voice use patterns than both healthy singers and singers with PVH. Future work should investigate the relationships between personality and vocal behavior in singers and nonsingers with NPVH as well as nonsingers with healthy voices in order to understand inherent personality-related influences that drive voice use in different populations. We envision that broadening our understanding on how personality is related to daily voice use in different populations will result in improved ability to tailor therapeutic decisions to the individual and improve early detection of risk.

## Conclusions

The first aim of this investigation found that singers with nodules have significantly higher levels of Social Potency (i.e., dominance and assertiveness) and significantly lower levels of Control (i.e., impulsivity) than matched-control singers with healthy voices, and this combination of traits discriminated singers with nodules from vocally healthy singers. All singers tended to score highly (i.e., at least 0.5 *SD* above the population mean) on Extraversion-related traits (specifically those related to social dispositions), high on the Neuroticism-related trait of Stress Reaction, and high on the Absorption scale. The average trait profiles of both groups suggest that singers in general might be more likely to engage in daily voice use that is potentially phonotraumatic (e.g., high speaking vocal doses) in daily life.

The second aim of this investigation confirmed that there are relationships between personality traits/facets and speaking voice use in daily life that are more obvious in vocally healthy singers. Singers without vocal pathology who had higher levels of traits related to happiness (i.e., Wellbeing) and dominance (i.e., Social Potency) and lower propensity to use caution (i.e., Harm Avoidance) tended to speak more (i.e., higher speaking vocal doses), more loudly (i.e., higher mean SPL), with more variability in intensity (i.e., wider SPL range and higher maximum SPL), with increased periodic energy (i.e., higher CPP), and with more abrupt/pressed glottal closure (i.e., lower mean *H*_1_ *–H*_2_), all of which could potentially increase the risk of phonotrauma.

## Appendix A

### Higher-Order and Facet Scales With Descriptions of High Scores From the Multidimensional Personality Questionnaire–Brief Form (Patrick et al., 2002; Tellegen & Waller, 2008)

**Table:**

Scale	Description/representative items
Positive Emotionality	Tendency to experience positive emotions; responds to active and rewarding environments
Wellbeing	Has a happy disposition; does fun things; optimistic
Social Potency	Enjoys visibility; dominant; persuasive; likes to be in charge
Achievement	Ambitious; enjoys effort; likes challenge; persistent; hard-working
Social Closeness	Sociable; warm, affectionate; has close relationships
Negative Emotionality	Tendency to experience negative emotions; prone to experience anxiety and anger
Stress Reaction	Nervous/tense; anxious; sensitive/vulnerable; guilt-prone; easily upset
Alienation	Feels betrayed, exploited, or mistreated; believes others want them to fail; feels unlucky
Aggression	Enjoys violence; vengeful; enjoys distressing others; victimizes others for own gain
Constraint	Tendency to inhibit impulse expression, unconventional behavior, and risk taking
Control	Cautious/careful; plans ahead; reflective; sensible; anticipates events
Harm Avoidance	Avoids risk of injury; dislikes dangerous emergencies; dislikes risky adventures
Traditionalism	Advocates high moral standards; endorses religion; endorses strict child rearing; opposes permissiveness
Absorption	Can imagine vividly; engrossed in own thoughts; responsive to evocative stimuli; responsive to involving stimuli; thinks in images

*Note.* Absorption is a primary trait (i.e., facet) scale that does not load on to any of the three higher-order scales.

## Appendix B

### Group-Based Means (Standard Deviations) for Weeklong Ambulatory Speaking Voice Distributional Characteristics of Vocal Dose, Sound Pressure Level (SPL), Neck Surface Acceleration Magnitude (NSAM), Fundamental Frequency (f_o_ ), Cepstral Peak Prominence (CPP), and the Difference Between the First Two Harmonic Magnitudes (H _1_ –H _2_)

**Table:**

Ambulatory voice parameter	Vocally healthy singers	Singers with nodules
Vocal dose		
Phonation time (%)	5.88 (2.11)	8.12 (1.94)
Cycle dose (cycles/hr)	49,268 (17,787)	68,315 (17,857)
Distance dose (m/hr)	190.90 (95.70)	303.15 (113.82)
SPL (dB SPL)		
*M*	83.44 (5.59)	86.84 (5.33)
*SD*	10.76 (2.63)	11.14 (2.57)
5%	66.08 (5.47)	68.13 (6.65)
95%	101.68 (8.76)	105.02 (7.68)
5%–95%	35.60 (8.81)	36.89 (8.72)
Skewness	.08 (.36)	−.13 (.33)
Kurtosis	3.22 (.54)	3.20 (.76)
NSAM		
Skewness	−.07 (.25)	−.17 (.25)
Kurtosis	2.94 (.35)	2.92 (1.61)
*f_o_*		
Mode	200.55 (17.67)	202.14 (18.31)
*SD*	60.86 (8.13)	58.30 (9.13)
5%	170.14 (13.00)	169.67 (13.21)
95%	345.22 (28.17)	337.62 (31.97)
5%–95%	175.08 (23.38)	167.95 (24.84)
Skewness	2.30 (.35)	2.16 (.50)
Kurtosis	12.39 (2.64)	11.98 (4.22)
CPP		
*M*	21.69 (2.14)	22.70 (1.43)
*SD*	4.24 (.54)	4.38 (.41)
5%	14.44 (1.09)	15.09 (.70)
95%	28.19 (2.55)	29.36 (1.61)
5%–95%	13.75 (1.70)	14.27 (1.25)
Skewness	−.15 (.23)	−.22 (.21)
Kurtosis	2.39 (.18)	2.42 (.15)
*H*_1_ *–H*_2_		
*M*	4.87 (2.55)	4.02 (2.32)
*SD*	6.62 (.70)	5.95 (.64)
5%	−4.16 (2.78)	−4.27 (2.79)
95%	17.55 (2.63)	15.19 (2.56)
5%–95%	21.71 (2.25)	19.45 (2.19)
Skewness	.66 (.21)	.66 (.27)
Kurtosis	3.58 (.55)	4.15 (.66)
